# Impact of Microstructural Anisotropy on the Low-Cycle Fatigue of S420M Steel

**DOI:** 10.3390/ma18102365

**Published:** 2025-05-19

**Authors:** Stanisław Mroziński, Grzegorz Golański, Karina Jagielska-Wiaderek, Arkadiusz Szarek

**Affiliations:** 1 Faculty of Mechanical Engineering, Bydgoszcz University of Science and Technology, 85-074 Bydgoszcz, Poland; stanislaw.mrozinski@pbs.edu.pl; 2 Faculty of Production Engineering and Materials Technology, Czestochowa University of Technology, 42-201 Czestochowa, Poland; grzegorz.golanski@pcz.pl (G.G.); k.jagielska-wiaderek@pcz.pl (K.J.-W.); 3 Faculty of Mechanical Engineering, Czestochowa University of Technology, 42-201 Czestochowa, Poland

**Keywords:** low-cycles fatigue, anisotropy, S420M steel

## Abstract

This paper presents the results of an analysis of the mechanical properties of the S420M steel samples collected both perpendicular and parallel to the rolling direction. The scope of the analysis included the following: a static tensile test, a hardness measurement, a low-cycle fatigue test, and a microstructure analysis of the analysed material. During the analysis, it was found that the rolling direction had little effect on the most important strength parameters determined in the static tensile test, but had a significant effect on the fatigue properties. During fatigue testing, a significant reduction in fatigue life (from 50% to almost 300%) was observed for samples perpendicular to the rolling direction. The largest reduction in fatigue life was observed at the ε_ac_ = 0.25% strain level (almost 300%), while the smallest was at ε = 0.25% (50%). A comparative analysis of the results of constant-amplitude and programmed fatigue tests confirmed the validity of using accelerated life tests to determine the low-cycle fatigue properties of construction materials. The results of the experimental verification of the Palmgren–Miner linear hypothesis of fatigue damage accumulation confirmed the significant influence of the material data on the results of fatigue life calculations.

## 1. Introduction

A prerequisite for fatigue life calculations of a structural element is knowledge of the fatigue properties of the material (fatigue graph) from which the structural element is made, and the loading program (load spectrum) [1,2]. The fatigue properties of rolled materials, e.g., steel and its alloys, are usually determined according to relevant standards, most often with the use of material samples oriented parallel to the rolling direction. During sample preparation, anisotropy resulting from non-uniformity in the steel microstructure is ignored. Based on the literature analysis, it can be concluded that structural inhomogeneity may impact the mechanical properties of metals [3,4,5]. Sometimes, structural inhomogeneity may be intensified by loads exceeding the permissible limits of structural elements [6,7,8,9]. Structural inhomogeneity may also result from manufacturing processes used in the production of construction materials, such as rolling, welding, and extrusion [10,11].

Based on an analysis of literature data [12,13], it can be concluded that studies of the influence of anisotropy on mechanical properties most often concern material samples collected from sheet metal with a thickness not exceeding 15 mm. The variation of anisotropy is obtained by changing the inclination angle of the sample relative to the rolling direction (0 < α < 90). In extreme cases, samples are obtained that are parallel (α = 0°) or perpendicular (α = 90°) to the rolling direction. The aforementioned analyses overlook the material properties in the transverse direction (i.e., the direction perpendicular to the sheet metal surface). Based on the literature analysis, it can be concluded that structural elements made of rolled steel with a thickness greater than 15 mm are characterised by worse mechanical properties in the direction perpendicular to the surface (through the thickness) compared to those obtained in the rolling direction. The authors are not aware of the test results for that sample orientation.

**Figure 1:**
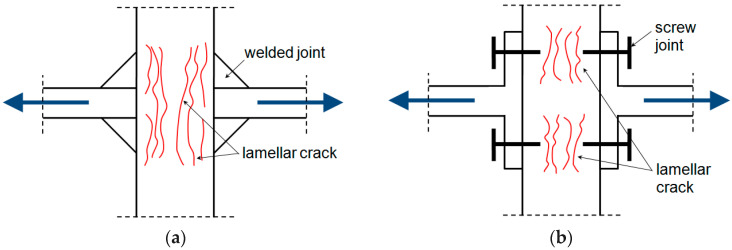
Scheme of lamellar cracks: (**a**) welded joints, (**b**) threaded joints.

Anisotropy of the mechanical properties of metal alloys (especially rolled ones) may contribute to the occurrence of the so-called lamellar cracks [14], especially in the case of welded or threaded joints (Figure 1).

That phenomenon is especially undesirable, particularly for structures where the elements are loaded with forces acting in the direction of the material thickness. It is most often the case, for example, with rigid, highly loaded welded structural nodes. For this reason, in certain industries, such as shipbuilding and construction, specialised guidelines have been established for the selection and testing of steel and its alloys [15,16]. The main cause of the cracks is the presence of elongated non-metallic inclusions, particularly those containing sulphur.

In cases where a structural solution involves a significant thickness of the element, such as a weld, additional material tests are recommended. One of these is the test conducted in accordance with [14], also known as the Z-tensile test. The test is one of the most popular methods to help avoid the occurrence of the aforementioned cracks. Due to the significance of the issue of lamellar crack formation, the latest edition of the normative document [14] recommends performing the Z-tensile test also for structural elements with nominal thicknesses in the range of 10–15 mm. The standard specifies minimum values for the reduction of area *Z* of the sample during static testing. Welded structural nodes, similar to the one shown in Figure 1, with thick walls and an operating temperature below 0 °C, are also prone to brittle fracture. Designing a structure resistant to brittle fracture involves primarily selecting a steel grade that guarantees adequate resistance to cracking at the expected operating temperature according to the principles presented in [17].

The operating loads on machine components are primarily variable, with a highly diverse course. The importance of the material fatigue issue has led to the inclusion in some standard documents of a recommendation to take fatigue properties into account when designing structural nodes [18]. The standard divides the fatigue life area into two: low-cycle fatigue and high-cycle fatigue. Low-cycle fatigue is assumed to cover a range up to approximately 10^4^ ÷ 10^5^ cycles. However, critical fatigue parameter values are not defined in the standard.

Based on the analysis of critical values of mechanical properties of the material for structural nodes included in the standard [14], it can be stated that the safety and reliability of structural elements subjected to variable loads during use have been limited only to taking into account material parameters determined during static load tests (Z-tensile test) as part of the design process. It is assumed that meeting the conditions set out in the standard releases the engineer from the obligation to check the fatigue properties of the steel in the direction perpendicular to the rolling direction.

The originality and novelty of the paper are connected with performing low-cycle fatigue tests on the research material—construction steel samples taken perpendicularly to the surface of the sheet metal—and comparing them with the results of tests conducted on the material—samples oriented parallel to the rolling direction. Currently, there are no publications presenting the results of fatigue tests of samples oriented perpendicularly to the surface of the sheet metal. This is most likely due to the difficulty in performing tests when the length of the measurement section of a specimen is small. The impact of sample orientation was evaluated based on the basic parameters of the hysteresis loop recorded during the tests, and the fatigue life obtained during the tests and calculations. The research problem addressed in this paper is the quantitative assessment of the impact of material sample orientation on fatigue properties. The scope of this work includes static and fatigue tests on S420M steel samples collected parallel to the rolling direction and perpendicular to the sheet surface. The obtained fatigue resistance test results for the material under study support the fatigue life calculations based on the determined material data.

## 2. Test Description

As part of the experimental research, a static tensile test (the Z test) was carried out in accordance with the requirements contained in the standard [14], a hardness measurement was performed in accordance with the standard [19], a low-cycle fatigue test was conducted on samples collected from the sheet metal, oriented parallel and perpendicular to the rolling direction, in accordance with [20], and microscopic observations we made. Towards the end of the paper, there is an experimental verification of the Palmgren–Miner linear hypothesis of fatigue damage accumulation [21,22].

The test specimens were collected from a 40-mm-thick steel sheet, the chemical composition of which was determined using a Bruker Q4 Tasman spark spectrometer (Bruker, Billerica, MA, USA) (Table 1). The chemical composition of the test material corresponds to that of the S420M steel.

### 2.1. Methodology of Static Tensile Tests

**Figure 2:**
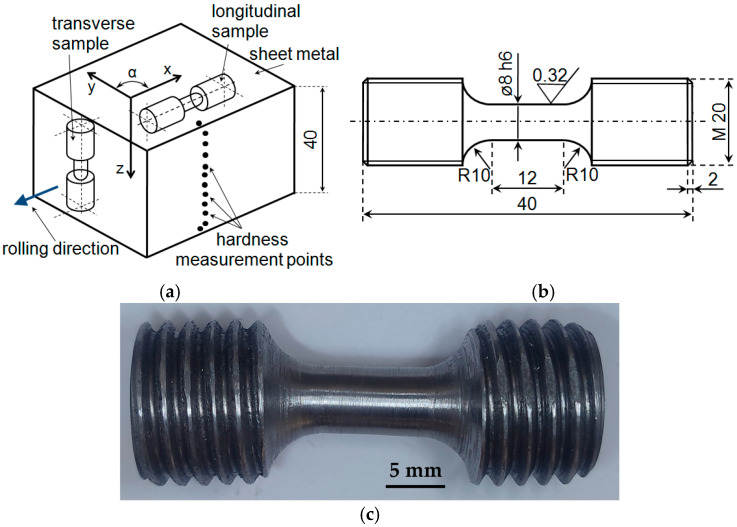
Samples for the static and fatigue tests: (**a**) orientation of samples on the sheet metal, where: x—longitudinal direction, z—transverse direction; (**b**) dimensions of test samples; (**c**) image of test samples.

The samples for the static and fatigue tests were prepared according to the guidelines in the standard [12]. The static tests were conducted using the Intron 8502 testing machine (Instron, High Wycombe, UK). To conduct a comparative analysis of the results obtained using the samples with different orientations in relation to the rolling direction, the geometrical features of the samples were assumed to be the same. Considering the thickness of the sheet metal (40 mm), the total length of the samples for static and fatigue tests corresponded to the sheet metal thickness. The dimensions of the samples for static tests, due to the length of the measurement section, and in accordance with the standard [14], fully corresponded to those of the samples recommended for low-cycle fatigue tests [19]. Figure 2a presents the position of the samples on the sheet metal, while Figure 2b shows the shape and dimensions of the test samples.

The sample preparation was preceded by a hardness measurement of the side surface of the sheet metal. The hardness tests were carried out on the cross-section perpendicular to the rolling direction, according to the diagram presented in Figure 2a. The tests were performed using the B-scale Rockwell method, in accordance with the requirements of the standard [20], with the use of the KP-15002P hardness tester (KABID-PRESS, Warsaw, Poland).

Static tensile testing was carried out following the requirements of the standard [14]. During the tests, the instantaneous values of the loading force and the elongation of the sample were recorded. The elongation of the sample was measured using a static test extensometer (type 2630-110) (Instron, High Wycombe, UK) with a 10 mm base attached to the measurement section of the sample, with a measuring range of +100%. The force was measured using a force gauge head (2518-113) (Instron, High Wycombe, UK) with a measuring range of ±100 kN. The tests were performed at a temperature of 20 °C. The breaking of the sample in the area of the measurement section was assumed as the criterion for the end of the static test. Three static tensile test samples were performed for each rolling direction. The static and fatigue tests were conducted using the Instron 8502 testing machine (Instron, High Wycombe, UK).

### 2.2. Low-Cycle Fatigue Tests

**Figure 3:**
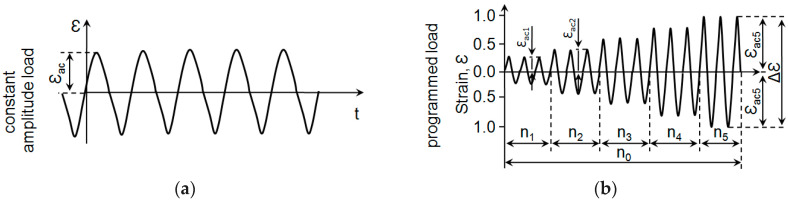
Test loading programme for the steel under consideration: (**a**) constant amplitude; (**b**) programmed.

The low-cycle fatigue tests consisted of subjecting the samples to a constant-amplitude load and a programmed load that gradually increased at a frequency of 0.2 Hz. Figure 3 and Table 2 show the variable loading programmes and their parameters, respectively.

During the constant-amplitude tests, instantaneous values of the force applied to the sample and the strain in the sample were recorded for selected load cycles. The force was measured using a dynamometer head (2518-113) with a measuring range of ±250 kN. The sample strain was measured using a static test extensometer (type 2630-110) with a 10 mm base attached to the measuring section of the sample, with a measuring range of ±10%. The criterion for the end of the fatigue tests was assumed to be the occurrence of deformation on the hysteresis loop arm in the compression half-cycle, in accordance with the standard [17]. For each level of strain, three constant-amplitude fatigue tests were performed. In the case of programmed loading (Figure 3a), 10 cycles were run at each of the five stages of the loading programme. After the completion of *n*_0_ = 50 cycles, the loading block was repeated until the permanent failure of the sample in the area of the measurement section. Whole blocks of the loading programme were recorded during the tests. Under programmed loading conditions, three fatigue tests were carried out for each sample orientation. The constant-amplitude and programmed tests were carried out at 20 °C. Both the constant-amplitude and programmed load tests were performed using the Intron 8502 strength testing machine.

### 2.3. Structure Analysis

The microstructure of the tested material was analysed using the Jeol JSM 6610LV (Tokyo, Japan) scanning electron microscope. Microstructure images were recorded at an accelerating voltage of 20 kV. The microstructural tests were performed using metallographic samples collected both perpendicular and parallel to the rolling direction. The samples were polished using 600#, 1000#, 1500#, and 2000# SiC sandpaper sheets, followed by three rounds of polishing with diamond spray. The samples were etched with a 4% solution of the reagent nital.

### 2.4. Fatigue Life Calculation

To highlight the influence of sample anisotropy on fatigue properties and, consequently, on fatigue life results, calculations were performed for the programmed loads implemented in this study. During the fatigue life calculation, the results of constant-amplitude tests performed on samples with different orientations were utilised in the form of fatigue graphs. The Palmgren–Miner linear hypothesis [19,20] was used to determine fatigue damage accumulation. The hypothesis assumes that in the case of constant-amplitude loading, each loading cycle contributes equally to the damage. This means that the fatigue damage, *D_i_*, is a linear function of the number of cycles, which can be expressed as follows:

(1) $$D_{i}=\frac{n_{i}}{N_{i}}$$

In the case of a multistage loading program used for the tests, cracking is to be expected if the condition is met.

(2) $$D_{i}=\lambda \sum_{i=1}^{k}\frac{n_{i}}{N_{i}}=1$$

where *λ*—the number of loading program block repetitions until fatigue failure, *k*—the number of the loading program stages, and *N_i_*—the number of cycles until fatigue failure at the *i*-th level.

## 3. Test Results and Analysis

### 3.1. Results of Static Tests

#### 3.1.1. Static Tensile Tests

**Figure 4:**
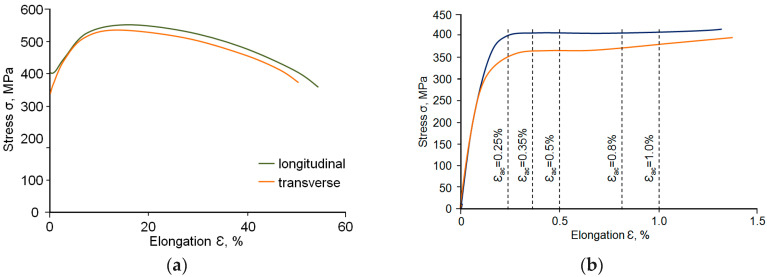
Tension curves: (**a**) tensile graphs, (**b**) levels of strain applied during low-cycle tests.

Figure 4a shows an example of static tensile curves. Table 3 shows the averaged results of the static test. Figure 4b, in turn, shows tensile curves for the strain range used in low-cycle fatigue testing.

**Figure 5:**
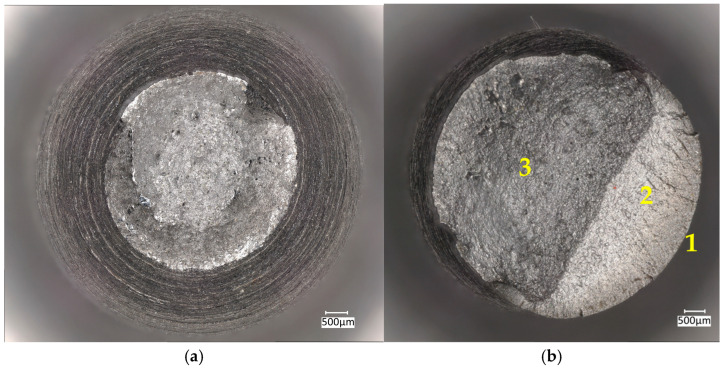
Macroscopic view of the sample: (**a**) after static tensile test; (**b**) after fatigue test, samples collected parallel to the rolling direction—1: fatigue focus, 2: fatigue zone, 3: brittle pit.

As expected, the strength parameters (*TS*, *YS*) of the samples collected parallel to the rolling direction are slightly higher than the values of the parameters determined for samples collected perpendicular to the rolling direction. Based on post-test measurements, a reduction in the plastic properties, i.e., reduction of the area (*RA*) and elongation (*El.*) was observed for the samples oriented perpendicular to the rolling direction. The obtained measurement results are presented in Table 3. A comparative analysis of the obtained results made it possible to conclude that the reductions in the *RA* are considerably greater than those required as per the standard. Thus, the steel meets the requirements set out in the standard [12] regarding the risk of lamellar crack occurrence. The break of the tested samples was macroscopic and plastic, preceded by clear plastic deformation in the smallest cross-section of the local deformation, resulting in the creation of a so-called neck (Figure 5a).

Due to the production method of thick sheet metal (controlled rolling and cooling) from construction steel with a ferritic–pearlitic structure, samples collected from the near-surface layer (Figure 2) are characterised by higher strength (YS, TS) and plastic (El., RA) properties—see Figure 4a and Table 3. This is mainly due to differences in grain size in the near-surface layers and in the central part of the thick sheet metal [23].

#### 3.1.2. Microstructure Analysis

**Figure 6:**
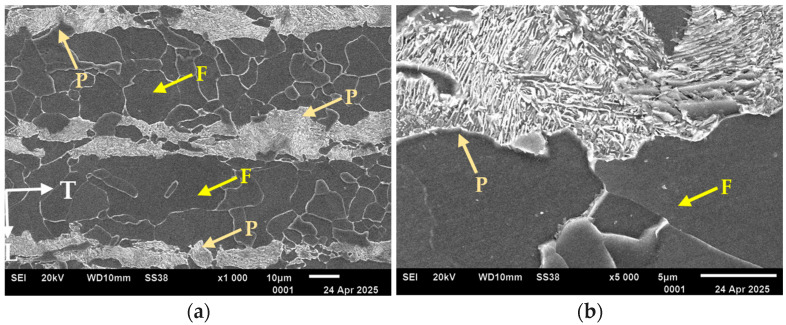
Structure of S420M steel: (**a**) banded steel microstructure, (**b**) pearlite in the tested steel, where: F—ferrite, P—pearlite, T—transverse direction.

The analysed alloy—S420M steel—was characterised by a banded ferritic–pearlitic microstructure with different grain sizes (Figure 6a). The volume share of ferrite and pearlite was similar throughout the total volume, at 68% and 32%, respectively. The grain size of ferrite, estimated using drawing standards [24], varied in cross-section I in the near-surface layer and was 10/11 (individual areas 8), while in the centre of the sheet metal, it was 9/8 for ferrite and 7 for pearlite. The pearlite was present in the form of separate colonies and was isolated at the ferrite grain boundaries in certain areas. A large dispersion of cementite was found in the pearlite (Figure 6b). Such a structure indicates accelerated cooling of the tested material after the plastic working process, which strengthens the material and achieves the required mechanical properties. In the case of construction steel with a ferritic–pearlitic structure, accelerated cooling after controlled rolling makes it possible—by fragmenting the microstructure—to obtain troostite (fine pearlite) and to partially slow down the recrystallisation process. This enables the significant strengthening of ferritic–pearlitic steel, achieving a yield strength of up to 500 MPa [23].

#### 3.1.3. Results of Hardness Measurements

**Figure 7:**
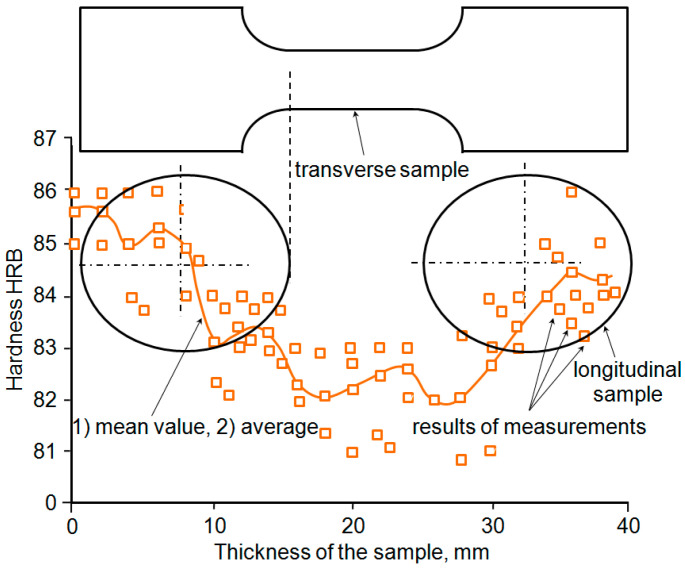
Rockwell HRB hardness distribution across the cross-section of the sheet metal.

The results of the performed hardness measurements are summarised in Figure 7. Based on the analysis of the hardness measurement results, it can be concluded that there is some variation in the hardness values in the cross-section of the sheet metal; the impact of the material thickness on the obtained hardness value is noticeable. In the inner section, the sheet is characterised by a slightly lower hardness compared to that in the near-surface layers. Considering the position of the sample on the sheet metal, it can be concluded that the measurement section of the samples perpendicular to the rolling direction is located in the part of the sheet metal with the lowest hardness. The hardness of the parallel sample was the same along its entire length. Considering the position of the samples on the sheet metal, it can also be concluded that the hardness was slightly higher than that of the measurement section of the perpendicular sample. This explains, to some extent, the higher strain values of the perpendicular sample at the same tension levels compared to those of the parallel sample observed in the tensile diagram (Figure 4).

The cooling of thick sheets after the controlled rolling process is not uniform throughout the entire volume of the material. As is known, faster cooling occurs near the surface of the sheet, while slower cooling is observed in its centre. The effect of different cooling rates across the cross-section of the sheet thickness results in minor differences in the microstructure of the material (grain size, cementite dispersion in pearlite), which translate into slight differences in mechanical parameters (Table 3), including hardness (Figure 7). Similar differences in hardness values in the cross-section of the analysed material, differing slightly in its volumetric microstructure, were also observed in a previous study [25].

### 3.2. Constant-Amplitude Low-Cycle Fatigue Tests

**Figure 8:**
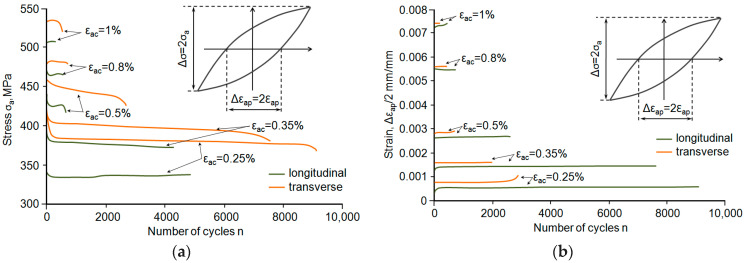
Hysteresis loop parameters as a function of the number of cycles: (**a**) stress amplitude *σ_a_*, (**b**) plastic strain amplitude *ε_ap_*.

The results of the constant-amplitude low-cycle tests were compiled according to the procedures specified in standard [17]. In the paper, two hysteresis loop parameters were assumed to analyse the test results, namely, the amplitude of stress *σ_a_* and the amplitude of plastic strain *ε_ap_*. Stress, in general terms, *σ*, in the sample was calculated as the ratio of the instantaneous force loading the sample to its initial cross-sectional area. Based on the analysis of the recorded hysteresis loops, it was found that the low-cycle properties of steel depend on the level of strain. For total strain of *ε_ac_* < 0.5%, steel strengthens slightly regardless of the sampling direction. This is confirmed by a reduction in stress *σ_a_* and a slight increase in the cyclic plastic strain range Δ*ε_ap_* over successive loading cycles. For comparison purposes, Figure 8 illustrates example changes in stress *σ_a_* and plastic strain *ε_ap_* as a function of the number of loading cycles for both sample orientation cases. For total strain *ε_ac_* > 0.5%, the situation is the opposite.

Analysis of the graphs (Figure 8) allows us to conclude that during cyclic loading, the *ε_ap_* strain of samples collected perpendicular to the rolling direction is greater than the value of that parameter for the samples oriented parallel (Figure 8b). In turn, the second analysed parameter (*σ_a_*) of perpendicular samples is smaller than the value of that parameter obtained for parallel specimens (Figure 8a). The above results apply to all strain levels. Due to the lack of a clear stabilisation period, the hysteresis loop parameters for further analyses were determined based on the period corresponding to half-life (*n*/*N* = 0.5). The values of stress *σ_a_* and strain *ε_ap_* obtained at individual strain levels were approximated by the equation proposed in the study [19], as follows:

(3) $$lg\;\sigma_{a}=\mathrm{lg}K+n^{\prime }\;\mathrm{lg}\varepsilon_{ap}$$

For the analytical description of a correlation between stress in general terms, *σ*, and strain in general terms, *ε*, the equation proposed by Ramberg–Osgood was used [26]:

(4) $$\varepsilon =\frac{\sigma_{a}}{E}+{\left(\frac{\sigma_{a}}{K^{\prime }}\right)}^{\frac{1}{n^{\prime }}}$$

**Figure 9:**
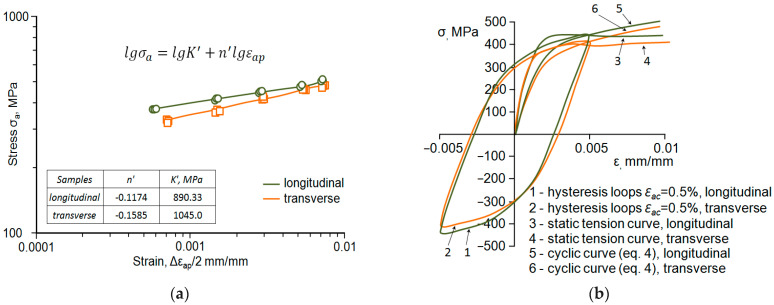
Diagrams of low-cycle fatigue test results: (**a**) cyclic graphs, (**b**) cyclic and static graphs.

Figure 9a shows the graphs described by Equation (1), while Figure 9b shows the graphs described by Equation (2) against the static tensile graphs.

The values of the hysteresis loop parameters, i.e., *σ_a_* and *ε_ap_* obtained at five strain levels from the *n*/*N* = 0.5 fatigue life are described by Equation (3) and shown in Figure 8a. In contrast, Figure 8b shows examples of hysteresis loops obtained at ε_ac_ = 0.5% strain level and graphs described by Equation (4) against the background of static tensile graphs.

The relative position of the static and cyclic strain curves confirms the impact of strain level on the low-cycle properties observed in Figure 8. For total strain *ε_ac_* < 0.5%, the cyclic curves are located below the static curves, which indicates weakening of the steel, while for total strain *ε_ac_* > 0.5%, the cyclic curves are located above the static curves, indicating cyclic strengthening of the steel.

**Figure 10:**
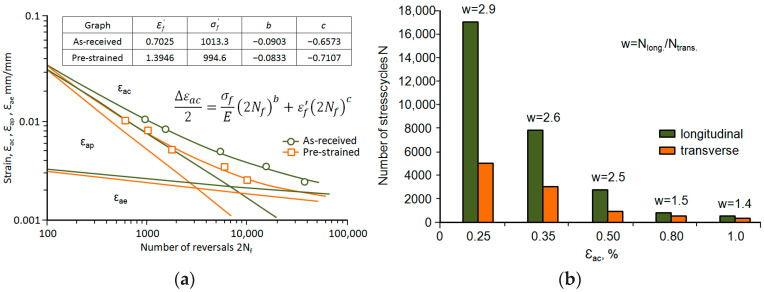
Diagram of fatigue life of the investigated steel: (**a**) fatigue curves, (**b**) column chart.

The fatigue life measurement results are shown in Figure 10 in the 2*N_f_*-*ε* coordinate system using the Manson–Coffin–Basquin equation [27,28,29] as follows:

(5) $$\frac{{∆\varepsilon }_{ac}}{2}=\frac{{∆\varepsilon }_{ae}}{2}+\frac{{∆\varepsilon }_{ap}}{2}=\frac{\sigma_{f}}{E}\;({2N_{f})}^{b}+\varepsilon_{f}^{\prime }({{2N}_{f})}^{c}$$

For comparison, Figure 10b shows the average fatigue lives obtained during the tests. Based on a comparative analysis of the position of the fatigue curves and the average lives, it can be concluded that the fatigue life of samples collected perpendicular to the rolling direction is significantly lower than the fatigue life of the samples oriented parallel to the rolling direction.

The fatigue samples were destroyed in a brittle manner, exhibiting no macroscopic plastic deformations, and displaying the formation of areas characteristic of that type of damage—fatigue focus, fatigue zone, and brittle pit (Figure 5b).

### 3.3. Programmed Low-Cycle Fatigue Tests

**Figure 11:**
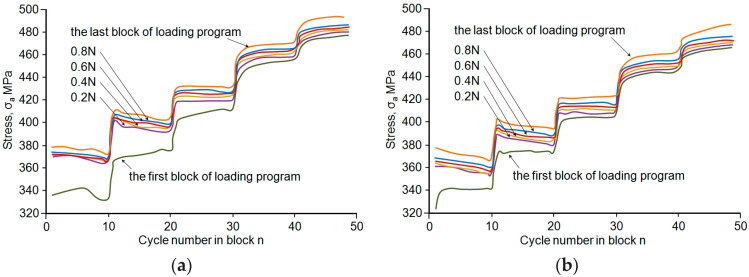
Stress *σ_a_* in the loading programme block: (**a**) parallel samples, (**b**) perpendicular samples.

The effect of strain level on low-cycle properties was also observed under the programmed loading conditions. To illustrate the changes in cyclic properties under programmed loading conditions, Figure 11a,b show multiple curves of stress *σ_a_* in subsequent repetitions of the loading program block for samples oriented parallel and perpendicular to the rolling direction.

**Figure 12:**
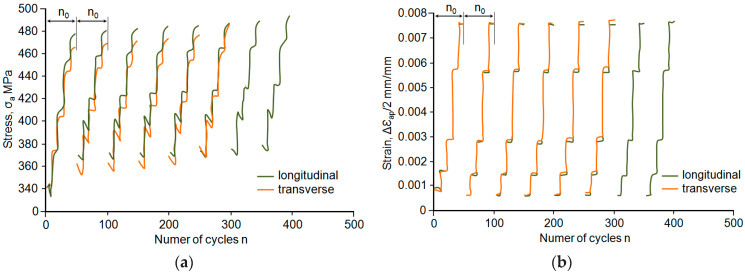
Loop parameters depending on the number of cycles: (**a**) stress *σ_a_*, (**b**) plastic *ε_ap_*.

Based on the graphs in Figure 11a,b, it can be concluded that after changing the strain range to *ε_ac_* < 0.5%, the stress *σ_a_* decreases. This pattern of stress change indicates a weakening of the steel, while at the *ε_ac_* > 0.5% level, stress *σ_a_* increases slightly, indicating a strengthening process of the steel. It should be emphasised that, during the performance of 10 cycles per stage, no stabilisation occurs due to the subsequent strain change at the following stage of the programme. The processes observed in subsequent repetitions of the loading programme block take place at higher levels of stress *σ_a_*. The above is illustrated in Figure 12. A comparative analysis of the hysteresis loop parameters at the same strain levels shows that stress *σ_a_* in the samples parallel to the rolling direction is higher than the corresponding stress in perpendicular samples. In the case of plastic, *ε_ap_*, in perpendicular samples, it was higher than that observed in parallel samples.

The above confirms the results of constant-amplitude tests, during which, in the samples collected perpendicular to the rolling direction, at the same load levels, the strain was greater than the strain in the case of the samples oriented parallel to the rolling direction.

**Figure 13:**
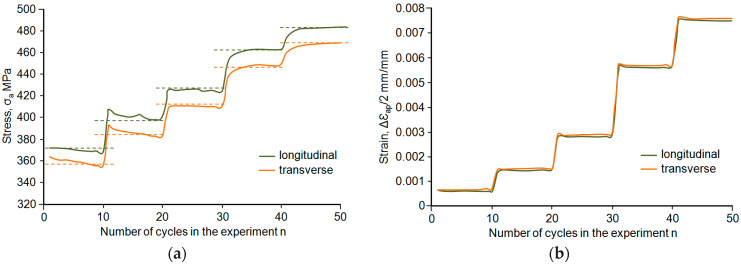
Loop parameters in the perpendicular sample programme block: (**a**) stress *σ_a_*, (**b**) plastic *ε_ap_*.

To compare the impact of sample orientation on the cyclic properties of steel, Figure 13 shows the changes in the amplitude of stress *σ_a_* and the amplitude of plastic *ε_ap_* for the loading programme blocks corresponding to half of the fatigue life of the parallel and perpendicular samples. Figure 13a additionally shows the stress levels (*σ_a_*) observed at half fatigue life (*n*/*N* = 0.5) at the same total strain levels *ε_ac_* during the constant-amplitude tests. Based on a comparative analysis of the position of the stress *σ_a_* and plastic *ε_ap_* data, it can be stated that during programmed and constant-amplitude loading, the cyclic properties obtained at individual strain levels for the same periods are comparable. The values of the hysteresis loop parameters (*σ_a_*, *ε_ap_*), shown in Figure 13, were the basis for the preparation of the graph in Figure 10.

The test results shown in Figure 13 confirm the validity of using low-cycle accelerated life tests to determine the material properties. These tests involve using a single sample and subjecting it to a gradually increasing load. The test method was described and verified in studies [30,31,32,33], inter alia.

As in the case of the constant-amplitude tests, the fatigue life of samples perpendicular to the rolling direction under programmed loading conditions was lower than that of the samples parallel to the rolling direction. The average fatigue life results are summarised in Table 4.

The fatigue life results presented in Table 4 show that the *N_obl_* fatigue life of the samples perpendicular to the rolling direction, calculated using the material data determined for the parallel samples (*N_obl_* = 610), is significantly greater than the actual fatigue life of perpendicular samples (*N_Exp_* = 340). The obtained calculation results are almost 100% greater than the test results. It can be said that the obtained calculation results are in the “dangerous” zone of permanence.

A significant improvement in the consistency between calculation and test results was achieved by using the specific material data during permanence calculations. Although the calculation results are still in the “dangerous” zone (N_obl_ > N_Exp_), the consistency of permanence results obtained in the calculations and tests is significantly higher. The above proves the usefulness of the Palmgren–Miner hypothesis in the area of variable loads under study. The satisfactory consistency between the calculation and test results can be explained by the small number of repetitions of the loading programme block during the testing and calculations. The above has been confirmed multiple times in other studies [34,35,36].

Due to the significant influence of sample orientation on fatigue life, when selecting rolled steel for the structural elements of considerable thickness, in addition to the classic static tests described in the standard [14], fatigue tests should also be performed using samples oriented perpendicular to the rolling direction. Failure to take the above into account may (despite meeting the standard conditions) lead to the occurrence of fatigue cracks before the expiry of the fatigue life calculated using the data determined for the samples parallel to the rolling direction and, consequently, result in structural failure.

The obtained results are influenced by the structure of the steel considered in the two directions, with ferrite and pearlite bands oriented either perpendicular or parallel to the direction of the tensile force.

Following the analysis of the steel hardness, it can be concluded that the samples perpendicular to the rolling direction are characterised by a change in the hardness value along their length (Figure 9). The lowest hardness occurs in the measuring section of the sample, whereas for parallel samples, the material has a comparable hardness along the entire length of the sample. The presence of a more uniform plastic strain in the sample parallel to the rolling direction, compared to a transversely oriented sample, had a favourable effect on fatigue resistance. Crack initiation in the samples takes place in a similar way; however, it occurs earlier in the transverse sample due to the location of greater strains in the sample perpendicular to the rolling direction. This applies to both constant-amplitude and programmed loading. This study shows that designers should pay attention to the selection of mechanical properties when modelling or calculating the details of structural elements.

## 4. Conclusions

The purpose of this study was to assess the impact of the microstructural anisotropy of S420M steel on its mechanical and fatigue properties. The samples were collected from the S420M steel sheet, parallel and perpendicular to the rolling direction. The mechanical and fatigue properties were determined under static and variable loading conditions. The following conclusions can be drawn based on the performed tests:

1. The direction of collecting samples from the sheet metal has little impact on the basic strength parameters, i.e., TS and YS, obtained in the static tensile test.
2. A comparative analysis of the reduction of area RA and the elongation of area El. shows a slight drop in these parameters in the case of perpendicular samples compared to parallel ones. Despite the reduction in the values of the RA and El. parameters, the S420M steel meets the material requirements as per the standard [14] related to the risk of lamellar crack occurrence.
3. The rolling direction has a significant impact on the fatigue properties. The fatigue life of samples oriented perpendicular to the rolling direction is shorter than the fatigue life of the samples oriented parallel to the rolling direction. The above applies to both constant-amplitude and programmed loading.
4. The comparative analysis of fatigue properties under constant-amplitude and programmed loading conditions indicates that the stabilisation course of cyclic properties under constant-amplitude and programmed loading conditions shows qualitative similarity regarding the nature of cyclic property changes and quantitative similarity regarding the values of hysteresis loop parameters during the same life periods.
5. The experimental verification of the Palmgren–Miner hypothesis showed a significant influence on the durability of the material data used for the calculations. The basis for the effectiveness of fatigue life calculation results is having material data determined under conditions reflecting the actual loading conditions of the structural element.

## Figures and Tables

**Table 1:** Chemical composition of the material used for the tests, %mass.

C	Si	Mn	P	S
0.16	0.22	1.53	0.018	0.009

**Table 2:** Constant amplitude and programmed load parameters.

No.	*ε_aci_*, %	*n_i_*
1	0.25	10
2	0.35	10
3	0.50	10
4	0.80	10
5	1.00	10

Programmed loading: n_1_ = n_2_ = n_3_ = n_4_ = n_5_ = 10 cycles; n_0_ = 50 cycles; R = −ε_min_/ε_max_ = −1; f = 0.2 Hz.

**Table 3:** Strength and plastic properties determined in the static tensile test for samples collected parallel and perpendicular to the rolling direction.

No.	Sample	TS	YS_L_	YS_H_	E	El.	RA
		MPa				%	
1	transverse	510	350	360	210	50	61
2	longitudinal	540	410	415	202	58	69

**Table 4:** Average fatigue life results from calculations and tests.

No.	Sample Type	Tests of Fatigue Life N_Exp_	Calculations of Fatigue Life N_Obl_	*N_Exp_*/*N_Obl_*
1	perpendicular	340	359	0.94
2	parallel	530	610	0.87

## Data Availability

The original contributions presented in this study are included in the article. Further inquiries can be directed to the corresponding author.

## Abbreviations

The following abbreviations are used in this manuscript:

*i*	Loading program block repetition
*k*	Number of loading program stages
*n_o_*	Number of cycles in the loading program block
*N_i_*	Number of cycles until fatigue failure at the i-th level
*λ*	Number of loading program block repetitions until fatigue failure
*E*	Young’s modulus
*El.*	Elongation
*K*′	Cyclic strength coefficient
*RA*	Reduction of area
*n*	Current number of cycles of constant-amplitude loading
*n*′	Cyclic strain hardening exponent
*N_f_*	Number of cycles to failure
*2N_f_*	Number of reversals to failure
*σ*	Stress in general terms
*σ_a_*	Amplitude of stress
*ε*	Strain in general terms
*ε_ac_*	Amplitude of total strain
*ε_ap_*	Amplitude of plastic strain
*ε_ae_*	Amplitude of elastic strain
Δ*ε*	Cyclic total strain range
Δ*ε_ap_*	Cyclic plastic strain range
Δ*σ*	Cyclic stress range
*ε_f_*′	Coefficient of cyclic plastic strain
*σ_f_*′	Fatigue strength coefficient
*b*, *c*	Exponents of strain diagram of fatigue
*TS*	Tensile strength
*YS*	Yield strength
YS_H_	Upper yield strength
YS_L_	Lower yield strength
